# Pediatric Rheumatic Fever With Acute Fulminant Carditis: A Case Report

**DOI:** 10.7759/cureus.47226

**Published:** 2023-10-17

**Authors:** Caroline Willaert, Sophie Lecomte, Nicolas Arribard, Montserrat Sierra-Colomina

**Affiliations:** 1 Pediatrics, Academic Children Hospital Queen Fabiola, Université Libre de Bruxelles, Brussels, BEL; 2 Pathology, CHU Brugmann, Université Libre de Bruxelles, Brussels, BEL; 3 Pediatric Cardiology, Academic Children Hospital Queen Fabiola, Université Libre de Bruxelles, Brussels, BEL; 4 Pediatrics/Pediatric Intensive Care, Toulouse Children's University Hospital, Toulouse, FRA

**Keywords:** group a β-hemolytic streptococcus pyogenes, infant, acute carditis, acute rheumatic fever, child

## Abstract

Acute rheumatic fever (ARF) is a multi-system inflammatory autoimmune disease. It is a significant cause of heart disease and early death worldwide, especially in children in developing countries. We present a case of acute fulminant rheumatic carditis in a child with no obvious predisposing factors, who resided in a developed country where this disease is not endemic. After pathological examination, a diagnosis of ARF with pancarditis was confirmed. This disease was not suspected before the pathological examination because of its low prevalence in Belgium.

## Introduction

Acute rheumatic fever (ARF) is a major cause of heart disease and early death worldwide, especially among children in developing countries [1]. ARF is a multi-system inflammatory autoimmune disease that follows an infection caused by *Group A β-hemolytic Streptococcus* (GAS), typically in pharyngitis [2]. There is a latency period of about one to five weeks before the first symptoms appear [1]. During the first episode of ARF, the incidence of carditis ranges from 30-82% [1]. Carditis is the most important prognostic factor in ARF [3]. We present a case of acute fulminant rheumatic carditis in a child with no obvious predisposing factors, who hailed from a developed country where this disease is not endemic.

## Case presentation

A previously healthy 11-year-old girl with unknown previous medical history presented to the emergency department with a two-day history of fever, cough, chest pain, and dyspnea. She did not report any recent infection (including pharyngitis) and had not been exposed to any sick individuals. Her family was originally from the Philippines but she was born in Belgium and had not traveled abroad recently. The initial physical examination revealed dyspnea. The patient’s vital signs showed tachypnea, pulse oximetry of 78% on ambient air, tachycardia at 156 beats per minute without hypotension, and a temperature of 39.7 °C. Severe bronchospasm in the context of bilateral pneumonia was initially suspected and the treatment with bronchodilators (Ventolin) was started without any response.

**Figure 1:**
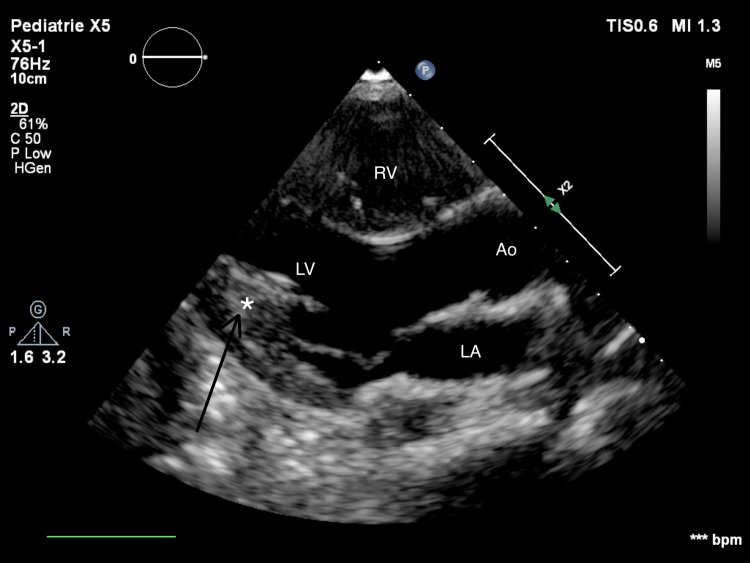
Cardiac ultrasound Hyperechogenic appearance of the myocardium (arrow)

The hypoxemic respiratory failure deteriorated despite providing non-invasive ventilation, necessitating intubation. The patient remained severely hypoxemic despite ventilation optimization. She developed severe hemodynamic instability with poor response to early resuscitation with fluid boluses and vasoactive drugs. Initially, biology tests showed compensated metabolic acidosis with hyperlactatemia, leukopenia with severe neutropenia without the associated inflammatory syndrome, and normal cardiac biomarkers. A nasal smear revealed an influenza A infection through polymerase chain reaction testing. A chest X-ray showed bilateral pulmonary edema. An electrocardiogram showed no abnormality. In this context, the differential diagnosis was cardiogenic shock secondary to fulminant viral myocarditis or secondary to acute respiratory distress syndrome due to influenza A infection. She received empirical antibiotic therapy with ceftriaxone and clindamycin, as well as antiviral treatment with oseltamivir. Inotropic support was maximized, hydrocortisone was initiated, ventilatory settings were increased, and inhaled nitric oxide was started before transferring the patient to the pediatric intensive care unit. The patient’s condition slightly improved. The cardiac ultrasound revealed heterogeneous myocardial dysfunction, without anatomical valve damage or valve insufficiency, and a hyperechogenic appearance (Figure 1).

**Figure 2:**
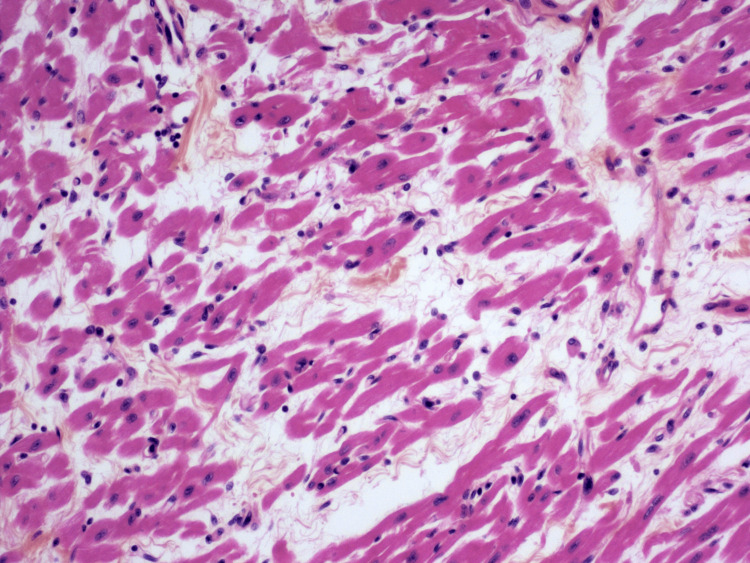
Myocardium The image shows the presence of some looser areas where the myocytes are not contiguous with edema and a discrete inflammatory lymphoplasmacytic infiltrate with some granulomatous-like formations

Cardiac enzymes remained within normal levels. In the next 24 hours, the patient progressed to a refractory cardiogenic shock. We then proceeded to peripheral venoarterial extracorporeal membrane oxygenation (ECMO), which was changed to central venoarterial ECMO in the following 24 hours due to flow limitations and distal limb perfusion complications. Post-surgery, the patient experienced disseminated intravascular coagulation and bleeding preventing her from achieving the required cardiac output to maintain end-organ perfusion. On the third day of hospitalization, she suffered an intracranial hemorrhage, which evolved into brain death. A pathological examination of the heart was decided to be performed after discussing it with the parents. Pathologically, the myocardium showed the presence of some looser areas where the myocytes were not contiguous with edema and a discrete inflammatory lymphoplasmacytic infiltrate with some granulomatous-like formations (Figure 2).

**Figure 3:**
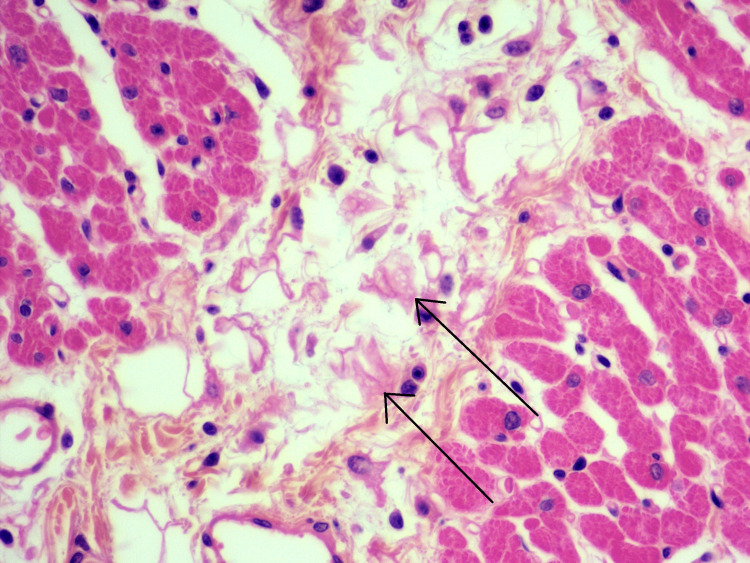
Fibrinoid-like necrosis The presence of a fibrinoid-like necrosis of the fibrous tissue was observed within these conjunctive spans (arrows)

**Figure 4:**
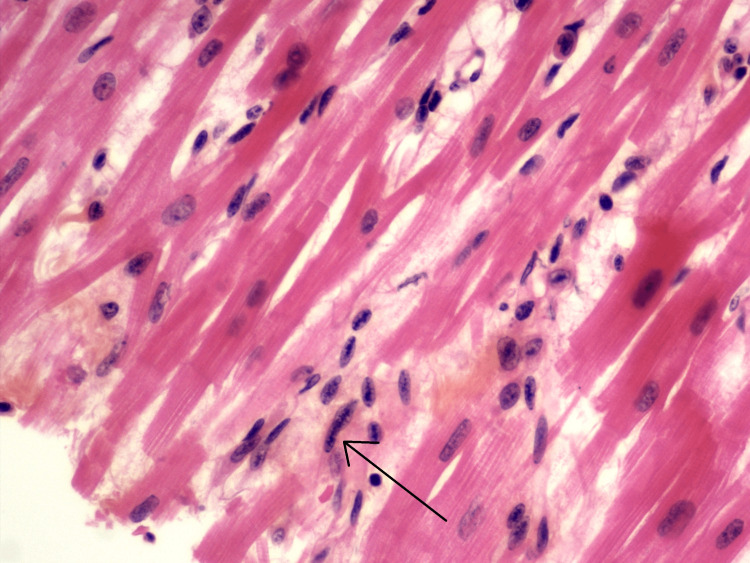
Anitschkow cells The presence of macrophages whose elongated nuclei showed an organization of the chromatin in a central band was observed within these conjunctive spans (arrow). These cells are also called Anitschkow cells. The granulomatous-like formations are also called Aschoff's nodules. We noted the absence of cardiomyocyte necrosis

This infiltrate was more pronounced around the conjunctive spans bordering the vessels. The presence of a fibrinoid-like necrosis of the fibrous tissue was observed within these conjunctive spans (Figure 3), and macrophages whose elongated nuclei showed an organization of the chromatin in a central band (Figure 4).

These cells are also called Anitschkow cells. The granulomatous-like formations are also called Aschoff’s nodules. We noted the absence of cardiomyocyte necrosis at these sites. These lesions are diffuse in the myocardium and do not have a left or right predominance. The rest of the examination ruled out catecholamine or toxic heart disease, global acute myocardial infarction (NB: the minimum delay for the histological sign of myocardial infarction is 12-24 hours), preexisting chronic or cardiac pathology, a metabolic pathology with cardiac repercussions, a viral, bacterial or parasitic infections, and particularly no endocarditis. The endocardium showed neither fibrous thickening nor inflammatory infiltrate. The valves showed a few areas at the connective level with the same image of necrosis described within the myocardial connective tissue. The cardionector tissue [atrioventricular node, the bundle of His (right and left branch), the sinus node, and some observed descending bundles from the sinus node to the atrioventricular node] did not show any specific lesions. In particular, no protein deposits were observed. The histological examination was compatible with cardiogenic shock in the context of ARF [4,5]. A discrete pericarditis was observed and correlated with the ECMO. After clinical, radiological, and pathological correlation, the diagnosis established was a cardiogenic shock in the context of probable ARF with pancarditis, exacerbated by a concomitant influenza infection.

## Discussion

ARF is a multi-system inflammatory autoimmune disease that is rare in the pediatric population [6]. The majority of pediatric rheumatic fever cases occur in children aged 5-14 years [7]. The incidence in school-aged children is 19 per 100,000 worldwide [6]. Its prevalence is higher in developing countries as the risk of GAS infection is greater. This is due to low socioeconomic status, overcrowding, malnutrition, poor hygiene, and lack of access to healthcare [2]. Susceptibility to develop ARF varies according to ethnicity and genetics (polymorphisms in several genes coding for immune proteins) [8].

Our patient’s family was originally from the Philippines where the incidence of the disease is one to four people per 1000 [8]. In Belgium, this condition is nowadays very rare [9]. According to several epidemiological and immunological studies, GAS has been clearly identified as the pathogen behind ARF. Molecular mimicry of this germ and autoimmunity are likely to play a major role in the pathogenesis of acute rheumatoid fever [9]. Recent studies have challenged the widely accepted concept that only a limited number of strains are capable of inducing rheumatic fever [10]. In our patient, there was not enough available blood postmortem to perform the biological tests required to prove a recent streptococcal A infection. The rise in the anti-streptolysin O and the anti-DNAse B titer could have been in favor of an untreated GAS pharyngeal infection (especially if there is an increasing trend in repeated readings) [1]. The evidence of GAS infection can also be confirmed by a positive throat culture for GAS or a positive rapid group A streptococcal carbohydrate antigen test in a child with clinical findings suggestive of streptococcal pharyngitis since GAS carriage occurs in 15% of healthy children [11].

The diagnosis of ARF is clinical as no biological diagnostic test can confirm this disease. The clinical standard for diagnosing ARF is based on the Jones criteria, last reviewed in 2015 by the American Heart Association. Our patient was included in the low-risk category (not from an endemic area, no recent clinical evidence of pharyngitis, and no known source of contagion) and met a major criterion (carditis) and a minor criterion (fever). This presentation fulfills the criteria of a possible ARF with a low index of suspicion. ARF has many different presentations that may vary geographically and by ethnicity. Carditis is the most common and the most serious manifestation [1]. This can affect the entire heart tissue, the condition known as pancarditis, with valvulitis being the most common presentation [8]. Carditis usually occurs within two to four weeks of GAS infection. Indolent carditis may also occur several months after the infection and is often the only sign of ARF. It ranges from mild subclinical involvement to severe carditis leading to acute heart failure [3]. Cardiac failure occurs in less than 10% of patients during the first episode of the disease [1]. Our patient presented with rapidly progressing heart failure, leading to refractory cardiogenic shock requiring mechanical circulatory support. Endomyocardial biopsy is considered the gold standard for the diagnosis of fulminant myocarditis [12]. In this case, the postmortem pathological examination revealed Aschoff’s nodules in the myocardium and confirmed the diagnosis of ARF [5], which had not been suspected given the lack of awareness about a previous GAS infection and the low prevalence of this pathology in Belgium.

The management of acute rheumatoid fever consists of controlling inflammation, managing carditis, eradicating GAS, and preventing recurrence [9]. Aspirin is the most commonly used anti-inflammatory medication. Naproxen may be a suitable alternative because of its similar efficacy with fewer side effects [9]. In cases of heart failure, standard therapy should be initiated [9]. However, in fulminant carditis, conventional vasoactive drug therapies and standard treatments for refractory heart failure and cardiogenic shock are of limited benefit. There is a significant accumulation of pro-inflammatory cytokines and circulatory disturbances that may require mechanical circulatory support, such as ECMO, and immunomodulators (high doses of glucocorticoids and immunoglobulins) [12]. There is a lack of high-quality evidence regarding the use of corticosteroids in carditis caused by ARF [13]. In cases of suspected ARF, pharyngeal eradication of GAS is important. In practice, penicillin G is commonly used. However, this depends on known resistance in different geographical areas [1]. Secondary prophylaxis by penicillin G is recommended in cases of cardiac involvement. The presence of rheumatic chronic heart disease is a determinant of secondary prophylaxis duration [1]. In countries where the risk of ARF is high, primary prevention is important. Belgium is not considered a significant at-risk country. Currently, the Belgian Antibiotic Policy Coordination Commission (BAPCOC) does not recommend the use of antibiotics in patients with acute sore throat. However, it does recommend antibiotic therapy in the following cases: at-risk patients (judgment on the basis of risk factors, history, and clinical assessment) and severely ill patients [14]. Our patient died of a brain hemorrhage, which is a side effect of ECMO, occurring in approximately 10% of cases [15]. In the literature, cases of fulminant carditis due to ARF, without evidence of valvular damage, are rare. Few such patients survive, despite ECMO support [16].

## Conclusions

Early recognition of cardiogenic shock secondary to fulminant carditis is essential. This case report highlights the importance of considering acute rheumatic fever as an etiology in fulminant carditis, even in the absence of risk factors such as recent pharyngitis or residing in an endemic location of the disease. The management must be carried out at four levels: the control of inflammation, the treatment of carditis with the use of hemodynamic support such as ECMO, the eradication of GAS, and the prevention of recurrence. It is highly advisable to request postmortem examinations of these patients in order to recognize emerging pathologies.
